# The Evaluation of Urban Community Health Centers in Relation to Family Physician and Primary Health Care in Southern Iran

**Published:** 2017-12

**Authors:** Mohammad Reza HEYDARI, Ahmad KALATEH SADATI, Kamran BAGHERI LANKARANI, Mohamma Hadi IMANIEH, Homataj BAGHI, Mohammad Javad LOLIA

**Affiliations:** 1. Deputy for Health, Shiraz University of Medical Sciences, Shiraz, Iran; 2. Dept. of Social Sciences, Faculty of Humanities & Social Sciences, Yazd University, Yazd, Iran; 3. Institute of Health, Health Policy Research Center, Shiraz University of Medical Sciences, Shiraz, Iran; 4. Dept. of Pediatrics, Shiraz University of Medical Sciences, Shiraz, Iran; 5. Shohadaye Valfajr Health Center, Shiraz University of Medical Sciences, Shiraz, Iran

**Keywords:** UCHC, FP, Primary health care, Ghaleo region, Shiraz

## Abstract

**Background:**

Shiraz University of Medical Sciences, Iran has developed a new version of health urban posts called Urban Community Health Center (UCHC) with primary aim of improving primary health care (PHC) in urban areas. The aim of present study was to evaluate this newly developed model of UCHC in Ghaleno, a suburban region of Shiraz, Iran in 2014.

**Methods:**

Besides descriptive analysis of foregoing model and considering its goal, plan, and dimensions, a qualitative study was carried out using in-depth interview with four managers of this model in Ghaleo region as well as the family physicians (FPs) of the model. Data were analyzed using conventional content analysis.

**Results:**

Evaluation of this model in 7-month period after implementation, showed that population coverage was raised from 23% to 84%, a remarkable and increasing achievement of 61%. The universal package is a protocol for providing healthcare services based on PHC.

The descriptive study of the model based on physicians view, explored real FP, population’s satisfaction, importance of physician’s assistant, and payment system reform in the model.

**Conclusion:**

Ghaleno model has unique objectives in providing healthcare services in urban areas. The findings of this study call for further evaluation, specifically in quality care services. The success and continuation of this model demand the support of policy makers.

## Introduction

Healthcare systems in the new millennium are faced with potential challenges (1) including socio-political (2–4), economic (5, 6), and medical aspects (7, 8). This is due to formation of complex system facing healthcare policy makers (9), who experience challenges namely delivering high quality and efficient, effective, and accessible health care services against a background of financial constraints and rising costs (10). Many potential challenges such as HIV, noncommunicable diseases (NCDs), mental health, and aging (11) in addition to regional problems, the main potential impediments are the health care rising costs, the main challenge existing even in industrialized nations (12–14). The costs are continuously rising, Medicare costs for the elderly may increase by six-fold in the year 2040 in contrast to 1987 (15). Due to its importance, targeting is considered as an efficient and cost-effective public health strategy (13), (16). Policy makers are looking into new models of primary care delivery to increase quality as well as productivity (17).

Two main approaches were considered to overcome the challenges that include PHC and FP, each having its own theoretical basis. In regard to PHC, it can reduce many challenges such as HIV (18), disabilities (19), hospitalization (20), and NCDs (21). In parallel, FP prioritizes and delivers care according to a broad agenda based on patient needs (22).

Iran healthcare system is faced with the same challenges chronically (23) as other healthcare systems around the world, including global and regional problems. In regard to growing population, increased urbanization, and broad social changes accompanied by considerable transformations most important challenges include the increasing rate of NCDs, aging, mental health, and AIDS (11). The healthcare system has undergone three main reforms in the past three decades. The first phase occurred during the mid-1980s with emphasis on PHC in rural regions based on declaration of Alma-Ata. This reform led to many outstanding achievements (11, 24). The second reform was creating family practice and referral system and universal health insurance in rural areas and small cities with populations less than 20000 that was implemented in 2005. The third reform called Health System Evolution Plan (HSEP) transpired in 2014 following the 11^th^ presidential election in Iran and policy changes in Ministry of Health and Medical Education (MOHME). Fars and Mazandaran Provinces are two pilot provinces that initiated customized FP scheme since 2012.

Fars Province has started new urban posts called Urban Community Health Center (UCHC) since Nov 30, 2014, as a creative activity with belief in PHC (11). These centers included customized model of urban health centers to deliver PHC in urban communities. Simultaneous to operation of these centers, another center was inaugurated in the Ghaleno county of Shiraz.

Ghaleno is a county in the east of Shiraz, Iran. According to last census, its population is 18397 people with 6034 families. Of these, 793 people are foreign nationals, mostly Afghans. In this region, there was only one urban post house, one healthcare subcontractor, and one supplementary post that provided healthcare. In 2012, two private FPs and two governmental FPs assigned by Shiraz University of Medical Sciences were engaged in FP project.

Since the principal motive of this model was to promote healthcare system based on FP and PHC, the main goal of present study was to evaluate all dimensions of the model and its objectives. Two main questions posed in this study were; 1. How the model was conducted and what are its dimensions. 2. What is the attitude of FPs involved toward the model?

## Materials and Methods

This is a study, which was done in 2014 in the Ghaleno region, a suburban region of Shiraz, Iran. This descriptive study attempted to address the questions associated with the characteristics of the population or situation under study. However, descriptive study did not intend to answer questions about how/when/and why such characteristics occurred (25). Therefore, this study only includes two descriptive parts.

At first, we evaluated all dimensions of the model for which we gathered all aggregated data in the defined IT system of the model. This aspect of descriptive analysis concerned all dimensions of the model specifically vaccination, IUD, Pup smear, oral health, environment and occupational health, nutritional consultation, psychologist consultation, outpatient services and if necessary referral system. Further defined processes included non-communicable diseases, nutrition, mental health, family and population health, communicable disease, health education, and environmental health units. Finally, statistical evaluation of data in family filing was carried out.

The second part consisted of a qualitative study based on conventional content analysis. For this reason, data gathering was done with structured interviews by 4 physicians who participated in the model. The questions raised in interviews, recorded digitally, included; are you satisfied with this model and its processes? Does this model lead to increasing workload of FP? Do you want to continue your cooperation? Is the population under your coverage satisfied with this new program?

Ethical issues considered and verbal consent of the participants was transcribed during interviews. Data were interpreted to explore condensed meaning units (brief meaning of the interpretation), sub-themes (initial abstracted concept that explored the related condensed meaning units), and themes (an abstracted concept in relation to some subthemes). Research validation was conducted through member check method with 35 participants and research team to ensure accuracy and accessibility of the themes. In addition, trustworthiness was observed during the study with maintaining subjectivity, reflexivity, adequacy of data, and sufficient interpretation strategies. The research was conducted gradually, using proper methods for data collection, analysis and report 36, and data analysis was done in a reflexive manner. Thus, exploring themes was done based on back and forth activity between meaning units, and other explored concepts. In addition, analysis was done by checking the themes with peer check.

This study was based on the ethical codes of the American Sociological Association (26) and 7th revision of the Declaration of Helsinki about research ethics (27).

## Results

Ghaleno model included the main objectives and components, human resources division of labor and universal package.

Ghaleno has the main objectives with the least waste of resources and sustainable health coverage as follows:

- Providing PHC in the context of FP. Although network system in Iran has had remarkable attributes, it nevertheless cannot respond to demands of big changes taking place over the past three decades. Even though policy-makers are aware of Iran’s need for reform in healthcare system, they do not agree on the policies. Changes must be performed in FP and PHC. Since the health care services in Iran are based on primary health care, the FP program development and its relationship to public health centers should maintain this platform. In this model, every FP and their assistants were considered as a health provider and each physician act as a health unit. After providing a suitable room(s) with adequate facilities and equipment, all health family files of the population covered by the physicians were transferred to his/her office and delivered to her/his assistant. Since then all the data should be processed by the assistant who transfers all the responsibilities of public health care of his/her population to FPs, but preferably under physician’s supervision. However, some public services, such as vaccination to maintain the cold chain and IUD and Pap smear should be provided by the governmental system in order to implement infection control program. Nutrition and mental health services, environment, occupational and dental health should only be provided by governmental health care center. Governmental health care staff that previously provided health care services should be supervised by FP and FP’s assistant and shift to programs, previously receiving less attention, such as those requiring assessment, community based and educational programs etc. In the past, the same staff provided these services, as well as acting as assistants. In this context the following programs are to be implemented:
- Shifting a part of public service to the private sector to provide a dynamic, efficient and workable model.
- Providing maximum health care coverage (over 90%)
- Increasing people’s accessibility to FP services, both in terms of time and place.
- Improving the cost-effectiveness of services based on customized payment formula including per capita, fee for service, and bonus payment.
- Providing organized referral system from level 1 to level 2 based on rationing system of services. After coordination with the level 2 of special medical centers, each patient who needs to be referred and if willing is visited through appointment system in these centers.

**Fig. 1:**
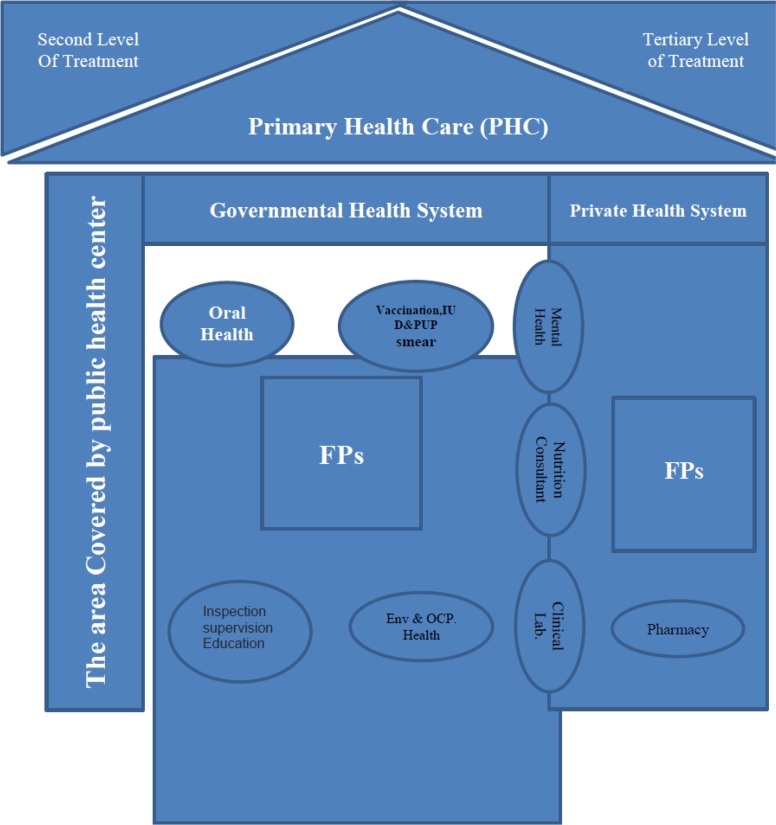
General design of Ghleno model; PHC in the context of FP

A general pattern of linkage between PHC and FP is required to achieve these objectives (Fig. 1). As the model shows both public and private systems in conjunction with network, system, and FP are active in this model. This model defines 7 main services which provide a comprehensive healthcare in level 1 alongside network system and preventive approach (Table 1).

**Table 1:** Dimensions of services defined in Ghaleno model

***Services***	***Dimensions***
Vaccination	Vaccination of target groups, according to national protocol
	Monitoring the cold chain
	Estimating and applying for required vaccines and sera
	Reporting side effects of vaccines
	Follow-up and serving vaccination of animals
IUD and Pup smear	According to national protocol and under FP supervision.
Oral health	Fluoride therapy target groups
	Sealants cylinder target groups
	Providing the necessary training to target groups and the community
	The first level of health services given in health centers
Environment and Occupational health	Inspection and monitoring centers for preparation and distribution of food by vendors
	Other health services beyond the scope of our joint environment
	Health workers in the region
Nutritional consultation	For target population
Psychologist consultation	For target population
Outpatient services and if necessary referral system	Attending outpatient visits by the family doctor and physical examination and referring those in need of examination by assistant based on national protocols
	Referring patients for the second level of services and appointment by the Secretary of State’s office or health center

The model considers both preventive and treatment dimensions with emphasis on prevention. Consequently, the most important aspect is comprehensiveness of the model. Our human resources and division of labor have been defined to achieve these objectives.

The staff recruited to perform in the program should meet the following requirements:

- FP: Qualified FP engaged in our model should hold general practitioner license, and received FP training since there is no specific educational field for FPs.
- FP Assistant: Bachelor or Master Degree in midwifery, health, family health, and nursing.
- Health attendants: Bachelor or Master Degree in midwifery or family health worked in these posts. They are assigned to 17 new tasks and work specifically as supervisors after starting the program.
- Nutritionist: Bachelor or Master degree in nutrition counseling for providing nutrition to patients or healthy people who need advice and referred to FP by public health centers.
- Psychologist: Bachelor or Master Degree in psychology who provides counseling for patients and healthy people that need advice and refer to FP by public health centers.
- Dentists: Holding doctorate degree in dentistry
- Vaccinator: Bachelor or Master degree qualified in midwifery or family health which performed vaccination, IUD and Pap smear before the implementation of primary health care.
- Occupational and Environmental Health staff: Associate graduated degree, Bachelor’s or Master’s degree in environmental and occupational health.

### Universal package

The universal package was designed to respond to the new health challenge, specifically NCDs and mental health, as a comprehensive approach since based on PHC in urban areas. The goal of this service pack is to promote health based on family-centered policy. This package includes processes that guide the FP and his/her assistant how to screen and continue providing services for the population. Table 2 shows seven defined units and their processes, which form the service package.

**Table 2:** Seven units defined for health delivery and related processes (Universal package processes)

***Unit part***	***Defined processes***
Non-communicable diseases	Diabetes screening process
	Caring for patients with diabetes
	Caring for patients with pre-diabetes
	Caring for patients with hypertension
	Caring for thalassemia couples / final suspect
Nutrition	Identifying pregnant women with growth delays
	Referral of patients with metabolic disorders to nutritionist
	Pre-diabetes care
	Educational programs by dietitian
Mental health	The screening process of patients referred by an FP assistant
	Caring for neurologic and mentally ill patients by doctor
	The care of mentally ill patients by psychologist
	Offering mental health services by a health care expert
Family and population health cares	Pregnant women
	Elderly
	Children
	Middle-aged
Communicable disease	Care and report of animal biting in the covered region
	Implementation of DOTS (Directly Observed Treatment, Short-course) protocol in family practice
	Vaccination of pregnant women and children under 6 yr by combined vaccination
	Summary, preparation and submitted Statistics of infectious diseases
Health education	Assessment of FP
Environmental health	Issuance of FP-related health cards

**Fig. 2:**
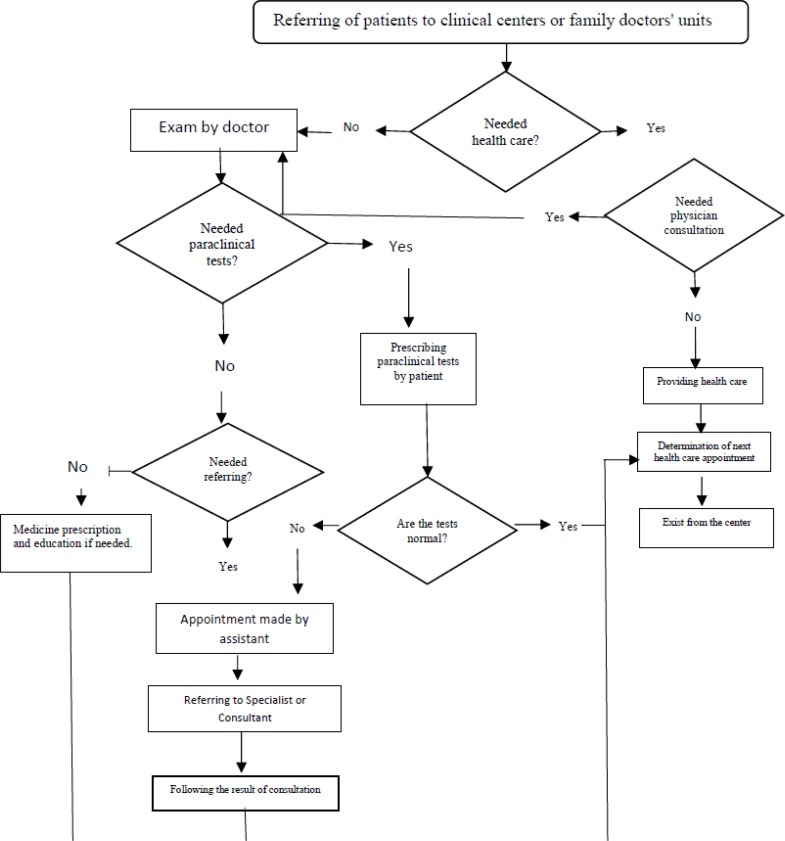
The process of diabetes screening in Ghaleno model

Universal package attempts to present a holistic model for health care delivery based on PHC and link with FP. Careful examination of the package clearly demonstrates that it is basically similar to declaration of Alma-Ata. Non-communicable diseases, mother and child health and environmental health constitute the foundation of universal package that secures the health of society. Any defined process such as diabetes screening (Fig. 2) is a guideline for FP and his/her assistant about the quality of service package.

### Evaluation

Considering the foregoing features, Ghaleno model started its operation on Sep 10, 2014, and was first evaluated after 7 months. Evaluating the model was carried out in order to assess the population coverage and doctor’s view using qualitative and quantitative methods. Concerning population coverage, the model achieved an important part of its goal (Table 3).

**Table 3:** First evaluation of Ghaleno model and family covered filing

***Doctor's name***	***The estimated number of households covered***	***The number of records delivered***	***Covered filing (before program)***	***The number of cases is made***	***Covered filing (after program)***	***Increasing percentage of coverage***
FP No.1	720	225	31	723	100	69
FP No.2	700	245	35	701	100	65
FP No.3	850	126	15	686	81	66
FP No.4	850	114	14	516	61	47
Total	3120	710	23	2626	84	61

Two FPs have reached and surpassed the intended target. They had 100% population coverage with more than 65% increase compared to before reform. This is a noteworthy achievement.

Qualitative evaluation of doctors’ view about Ghaleno model was done with semi-structured interviews. Data related to four main extracted themes were analyzed using conventional content analysis.

### Real FP

Real FP refers to the physician who stands by his real situation of families and works according to his/her actual obligations. All participants believed that they are real FPs because they access to all information about families, check them systematically, and treat them on time. Their duties were in line with FP’s protocol and all of them were satisfied with this model and their jobs.

### Population satisfaction

Considering the physicians’ perspectives, people were more satisfied with centralization of family health records and easier access to doctors. In addition, families were highly satisfied with all parts of care provided in the clinic.

Since I provide all of care in my clinic, families are more satisfied. Accordingly, greater numbers of families refer to my clinic as FP for registration rather than to private clinics. They want I add them into my population of DP

(FP No.2)

### The importance of assistance

Another theme of great importance is doctor’s assistance, which helps physician to collect family health information and categorize them. In addition, in some cases, assistant can help doctor to screen the patients.

Assistant plays the main role in reducing work pressure. Specifically, she helps with gathering data, providing families with primary health care and alleviates our concerns.

(Participant No.1).

An important point about assistant is that they have been educated according to the instructions of country in caring for pregnant women and infants.

Interestingly, our assistants are familiar with such instructions. They know how to screen and evaluate pregnant women and infants and refer them to appropriate centers whenever necessary.

(Participants No.2 and 4)

Moreover, the importance of assistance role demands their full-time employment.

### Payment system reform

Payment system needs reforming. The existing system is in conflict with equity. Therefore, health system should pay more attention to this concern, and respond appropriately about the salary of assistants.

## Discussion

Ghaleno model is similar to Croatia’s model where doctors and healthcare givers are educated and trained well in PHC domain. In addition, physicians are trained in higher level of healthcare delivery considering the key role of referral system. PHC in Croatia has a long history. It does not merely represent the patients’ gateway to the health care system, but it also provides a comprehensive level of health care (28). For designers of Ghaleno model, the significance in Croatia’s model was the privatization of health services. Croatia’s reform has led to important achievements (29).

Ghaleno model is also very similar to Tukey’s model that links PHC to FP. According to evaluation by WHO, Turkey’s model falls short of sufficient training of physicians and nurses, adequate salaries, and sufficient level of motivations in the staff (30). In Ghaleno, physicians have financial motivation, an issue deserving evaluation regarding other personnel engaged in health care system.

Thus, Ghaleno needs more emphasis on quality of care and on its sustainability, cost-effectiveness, and care givers satisfaction. Generally, Ghaleno model can be considered as a suitable model for dealing with new healthcare challenges. A case in point is that the FP by virtue of her his breadth of training in a wide variety of medical disciplines gains unique insights into the skills possessed by physicians in the more limited specialties (31). A conceptual model of this approach to FP and recent global health sector reforms leads to empowerment of FPMany studies show that FP is a cost-effective model that plays the main role in providing health care. In addition, FPs provide and coordinate care for a wide variety of patients’ problems, and prioritize them on the basis of relationships developed during multiple patient visits over time (22). FPs can be effective in managing acute illnesses, visit as opportunities to integrate care for specific diseases, mental health, and preventive care tailored to the specific needs of patients and families (22). On the other hand, proper PHC provides a favorable approach to overcome the new challenges to healthcare systems, a reason for creating a link between FP and PHC in our model.

In spite of these, Ghaleno has its own potential challenges in that doctors and their assistants lack academic training in FP program. According to the definition of FP, a physician practicing in this field must be educated and trained in the discipline of family medicine, care for people and do not just treat patients (31). In this context, none of our FPs has any education or training in family medicine. This is an important challenge in our model that is due to structural problems of medical education in Iran.

Although the model has had significant coverage an important point is, the quality of care monitored and evaluated in future studies. Another problem is that Ghalno model is in acute need of policy makers’ attention. In addition, healthcare and similar systems in Iran rarely receive due consideration, because of hospital-centered health care. On the other hand, healthcare policy makers in Iran are faced with wide-ranging and sometimes conflicting policies, a condition jeopardizing the future and sustainability of Ghaleno model.

Finally, there is a concern about privatization of this model. Because Iran is a developing country and its healthcare systems’ policies are continuously changing the privatization of this model imposes undue cost on the society, which is a problem related to weakness in management in developing countries. Although the model has moved well towards privatization, it may fail in long term. The private system seeks its own benefits that inevitably impede the progress of healthcare system. This is an important criticism about privatization network system of PHC.

## Conclusion

Shiraz University of Medical Sciences introduced two main changes in public health over the past four years, including FP program since 2012 and setting up UCHCs in 2014. These two modifications were based on their importance in health sector reforms. Ghaleno is a developed UCHC that includes private sector and cost-effective payment model. In medical domain, it refers to the potential capacities of FP and PHC. The universal package is a suitable model to encounter new challenges including NCDs and communicable diseases. The initial assessment of the model showed high rate of coverage. This model needs to be promoted about teamwork and quality of care. Simultaneously, this customized model deserves the rightful attention and support of policy makers.

## Ethical considerations

Ethical issues (Including plagiarism, informed consent, misconduct, data fabrication and/or falsification, double publication and/or submission, redundancy, etc.) have been completely observed by the authors.

## References

[1] Pescosolido BA Kronenfeld JJ (1995). Health, illness, and healing in an uncertain era: challenges from and for medical sociology. J Health Soc Behav, 5–33. 7560849

[2] Peterson S (2002). Epidemic disease and national security. Security Studies, 12:43–81.

[3] Hyams KC Murphy FM Wessely S (2002). Responding to chemical, biological, or nuclear terrorism: the indirect and long-term health effects may present the greatest challenge. J Health Polit Policy Law, 27(2): 273–91. 12043900 10.1215/03616878-27-2-273

[4] Judge K Platt S Costongs C Jurczak K (2005). Health inequalities: a challenge for Europe. UK Presidency of the EU, UK.

[5] Thomson S Foubister T Mossialos E (2009). Financing health care in the European Union: challenges and policy responses. WHO Regional Office for Europe, Copenhagen.

[6] Wang YC McPherson K Marsh T Gortmaker SL Brown M (2011). Health and economic burden of the projected obesity trends in the USA and the UK. Lancet, 378(9793):815–25. 21872750 10.1016/S0140-6736(11)60814-3

[7] Palatnik-de-Sousa CB Day MJ (2011). One Health: the global challenge of epidemic and endemic leishmaniasis. Parasites & Vectors, 4:197. 21985335 10.1186/1756-3305-4-197 PMC3214158

[8] Garrett L (2007). The challenge of global health. Foreign Affairs, 1:14–38.

[9] Plsek PE Greenhalgh T (2001). Complexity science: The challenge of complexity in health care. BMJ, 323(7313):625. 11557716 10.1136/bmj.323.7313.625 PMC1121189

[10] Buttigieg SC Gauci D (2015). Health Care Innovation Across Health Systems. In: Challenges and Opportunities in Health Care Management. Eds, Gurtner Soyez Springer, New York, pp. 47–60

[11] Imanieh MH Sadati AK Moghadami M Hemmati A (2015). Introducing the Urban Community Health Center (UCHC) as a nascent local model: Will it be a linchpin in the health sector reform in Iran? Int J Health Policy Manag, 4:331–332. 25905489 10.15171/ijhpm.2015.74 PMC4417642

[12] Reinhardt R Oliver WJ (2015). The Cost Problem in Health Care. In: Challenges and Opportunities in Health Care Management. Eds, Gurtner Soyez Springer, New York, pp. 47–59.

[13] Johnson SA (2010). Challenges in health and development: from global to community perspectives. Springer, USA.

[14] Fineberg HV Hunter DJ (2013). A global view of health—an unfolding series. N Engl J Med, 368(1):78–9. 23281981 10.1056/NEJMe1208801

[15] Newhouse JP (1992). Medical care costs: how much welfare loss? J Econ Perspect, 6(3):3–21. 10128078 10.1257/jep.6.3.3

[16] Ahokangas P Perälä-Heape M Jämsä T (2015). Alternative Futures for Individualized Connected Health. In: Challenges and Opportunities in Health Care Management. Eds, Gurtner and Soyez. Springer, New York, pp. 61–74.

[17] Simon B (2015). Group Medical Visits: Primary Care for the Next Century? Rethinking Choice Care. In: Challenges and Opportunities in Health Care Management. Eds, Gurtner and Soyez. Springer, New York, pp. 371–376.

[18] Joulaei H Motazedian N (2013). Primary health care strategic key to control HIV/AIDS in Iran. Iran J Public Health, 42(5):540–1. 23802115 PMC3684466

[19] Lishner DM Richardson M Levine P Patrick D (1996). Access to primary health care among persons with disabilities in rural areas: a summary of the literature. J Rural Health, 12(1):45–53. 10172606 10.1111/j.1748-0361.1996.tb00772.x

[20] Royal S Smeaton L Avery A Hurwitz B Sheikh A (2006). Interventions in primary care to reduce medication related adverse events and hospital admissions: systematic review and meta-analysis. Qual Saf Health Care, 15(1): 23–31. 16456206 10.1136/qshc.2004.012153 PMC2563996

[21] Puoane TR Tsolekile LP Caldbick S Igumbor EI Meghnath K Sanders D (2013). Chronic Non-Communicable Diseases in South Africa: Progress and challenges. South African Health Review 2012/13, South Africa.

[22] Stange KC Jaén CR Flocke SA Miller WL Crabtree BF Zyzanski SJ (1998). The value of a family physician. J Fam Pract, 46(5):363–8. 9597993

[23] Sadati AK (2017). Money Based Reform and Distorted Doctor-patient Interaction: A Critique of the Recent Health Sector Evolution Plan in Iran. Iran J Public Health, 46(4): 583–4. 28540282 PMC5439055

[24] Lankarani KB Alavian SM Peymani P (2013). Health in the Islamic Republic of Iran, challenges and progresses. Med J Islam Repub Iran, 27(1): 42–49. 23479501 PMC3592943

[25] Shields PM Rangarajan N (2013). A playbook for research methods: integrating conceptual frameworks and project management. USA, New Forums Press, USA.

[26] Association AS Association AS (1999). Code of ethics and policies and procedures of the ASA Committee on Professional Ethics. AAA, USA.

[27] Krleža-Jerić K Lemmens T (2009). 7th revision of the Declaration of Helsinki: good news for the transparency of clinical trials. Croat Med J, 50(2): 105–110. 19399942 10.3325/cmj.2009.50.105 PMC2681053

[28] Katiæ M Jureša V Oreškoviæ S (2004). Family medicine in Croatia: past, present, and forthcoming challenges. Croat Med J, 45: 543–9. 15495277

[29] Mastilica M Kušec S (2005). Croatian healthcare system in transition, from the perspective of users. BMJ, 331:223. 16037464 10.1136/bmj.331.7510.223 PMC1179777

[30] Ersoy F Sarp N (1998). Restructuring the primary health care services and changing profile of family physicians in Turkey. Fam Pract, 15(6): 576–8. 10078801 10.1093/fampra/15.6.576

[31] Rakel RE (2011). The Family Physician. In: Textbook of Family Practice. Eds, Rakel Rakel 8th ed, Elsevier Saunders, Chnina, pp. 3–16.

